# Acute Effects of Cocoa Flavanols on Blood Pressure and Peripheral Vascular Reactivity in Type 2 Diabetes Mellitus and Essential Hypertension: A Protocol for an Acute, Randomized, Double-Blinded, Placebo-Controlled Cross-Over Trial

**DOI:** 10.3389/fcvm.2021.602086

**Published:** 2021-03-15

**Authors:** Anouk Tanghe, Bert Celie, Samyah Shadid, Ernst Rietzschel, Jos Op ‘t Roodt, Koen D. Reesink, Elsa Heyman, Patrick Calders

**Affiliations:** ^1^Department of Rehabilitation Sciences, Ghent University, Ghent, Belgium; ^2^Univ. Lille, Univ. Artois, Univ. Littoral Côte d'Opale, ULR 7369 - URePSSS - Unité de Recherche Pluridisciplinaire Sport Santé Société, Lille, France; ^3^Faculty of Health Sciences, Sport, Exercise Medicine and Lifestyle Institute (SEMLI), University of Pretoria, Pretoria, South Africa; ^4^Department of Endocrinology, Ghent University Hospital, Ghent, Belgium; ^5^Department of Cardiology, Ghent University Hospital, Ghent, Belgium; ^6^Department of Internal Medicine, Ghent University, Ghent, Belgium; ^7^School of Cardiovascular Diseases (CARIM), Maastricht University Medical Centre, Maastricht, Netherlands

**Keywords:** type 2 diabetes, cocoa flavanols, vascular reactivity, blood pressure, muscular oxygenation, antihypertensive drugs

## Abstract

**Introduction:** Patients with type 2 diabetes mellitus are at high risk to develop vascular complications resulting in high morbidity and mortality. Cocoa flavanols are promising nutraceuticals with possible beneficial vascular effects in humans. However, limited research is currently available on the vascular effects in a diabetic population with inconsistent results. Possible reasons for this inconsistency might be heterogeneity in the given intervention (dose per time and day, single dose vs. split-dose, placebo formula) and the studied population (blood pressure at baseline, duration of diabetes, use of vasoactive antihypertensive and antidiabetic drugs, sex). Therefore, we aimed to develop a randomized, double-blinded, placebo-controlled cross-over trial to investigate whether cocoa flavanols have an acute impact on blood pressure and vascular reactivity in patients with type 2 diabetes with and without arterial hypertension.

**Methods and Analysis:** We will include participants in four groups: (i) patients with type 2 diabetes without arterial hypertension, (ii) patients with type 2 diabetes with arterial hypertension and 1 antihypertensive drug, (iii) non-diabetic participants with essential hypertension and 1 antihypertensive drug, and (iv) healthy controls. All participants will complete the same protocol on both testing days, consuming high-flavanol cocoa extract (790 mg flavanols) or placebo. Macrovascular endothelial function (flow-mediated dilation) and blood pressure will be measured before and after capsule ingestion. Forearm muscle vasoreactivity (near-infrared spectroscopy) and brachial artery blood flow (echo-doppler) will be assessed in response to a dynamic handgrip exercise test after capsule ingestion. Data will be analyzed with a random intercept model in mixed models.

**Clinical Trial Registration:** www.Clinicaltrials.gov, identifier: NCT03722199.

## Introduction

Nitric Oxide, produced by the endothelial cells, is of crucial importance for general vascular health. It triggers relaxation of the vascular smooth muscle cells through accumulation of intracellular cyclic guanosine monophosphate (cGMP) (1–5).

Type 2 diabetes (T2DM) is the most prevalent type [90% (6, 7)] of diabetes mellitus, a highly prevalent disorder [estimated at 425 million people worldwide in 2017 and is expected to be 629 million in 2045 (8)] and poses a challenge to global health. It is characterized by chronic hyperglycemia, which increases oxidative stress. Free radicals easily bind with and simultaneously deactivate nitric oxide to form peroxynitrite. Both high amounts of oxidative stress and nitric oxide depletion increase the risk for developing micro- (retinopathy, nephropathy, and neuropathy) and macrovascular (cardiovascular, cerebrovascular, and peripheral artery diseases) complications (9, 10). These complications decrease quality of life and increase the global burden of T2DM in terms of health care costs, morbidity [hypertension is present in >60% of all patients with T2DM (11)], and even mortality (12–15). The past years researchers have been investigating products to limit or delay the onset of diabetic vascular complications with special attention for non-pharmacological approaches to counter polypharmacy.

Flavanols are promising nutraceuticals from the flavonoid family, a class of polyphenols (16), which can be found in several fruits, beans, teas, red wine, but predominantly in cocoa products (16, 17). Especial interest goes to flavanols derived from the seeds of the cocoa bean (*Theobroma cacao*), cocoa flavanols (CF), as they have higher antioxidant activity and more phenolic compounds (18). The increased attention for the effects of cocoa originates from research on the Kuna Indians. In contrast to migrated Kuna Indians to urban areas, Kuna Indians living on the San Blas Islands off the coast of Panama show low blood pressure (BP), even with increasing age, and have lower frequency of diabetes mellitus, cancer, and cardiovascular diseases (19). Causal research for this cardiovascular protection focused on environmental factors including nutrition and revealed that island-dwelling Kuna drink daily more than five cups of cocoa with high concentrations of flavanols and procyanidins (19–21). Starting from evidence based on further research on the vascular effects of CF, the European Food Safety Authority (EFSA) stated that CF help to preserve endothelium-dependent vasodilation in healthy populations, if taken in quantities exceeding 200 mg CF daily. This equals 10 g high-flavanol dark chocolate or 2.5 g high-flavanol cocoa powder (22).

The mechanisms of action of these CF are still debated. It is believed that they improve endothelial function (23), decrease BP (24), ameliorate insulin sensitivity (25–27), influence various inflammatory processes (28), and prevent platelet aggregation (29, 30) *via* antioxidant properties (31, 32), increasing nitric oxide bioavailability (33, 34), and inhibition of the angiotensin-converting-enzyme activity (35, 36). These effects seem to be induced, at least in part, by epicatechin, a highly active monomeric form of CF (37, 38).

It could be presumed that populations with T2DM could benefit from the intake of CF as it potentially reduces cardiovascular risk. CF would indeed improve both endothelial function and insulin sensitivity and so influence cardiovascular as well as metabolic disorders (39). Nonetheless, limited research with a high degree of inconsistency due to large heterogeneity (dose per time or day, acute or chronic intake, single dose vs. split-dose, placebo formula, and characteristics of population e.g., sex, BP at baseline, and use of vasoactive drugs like insulin and antihypertensives) is reported.

As presented in Table 1, at the moment, six studies investigated the effect of chronic CF intake (8 weeks−1 year) on vascular function (40–45) in patients with T2DM. Only two showed a statistically and clinically relevant decrease in BP (40, 45) and only two indicated a statistically improvement of endothelial function (41, 42) (Table 1). The heterogeneity of results about mid- to long-term effects of CF in patients with diabetes had been recently approached through a systematic review and meta-analysis (48). This paper indeed shows low quality of evidence of slight improvements in BP after mid/long-term CF ingestion. However, risk of bias, imprecision of the publications, and inconsistency and heterogeneity among the reports are reported and could be cause for the lack of a definite conclusion. This meta-analysis ultimately highlights the need for further research with a robust methodology taking into account possible confounding factors like hypertension at baseline and intake of BP lowering medication. Antihypertensive drugs, which were never considered in these papers on chronic effects of CF in patients with T2DM, have indeed a great impact on vasoreactivity (49) and may hence interfere with the effects of CF.

**Table 1:** Characteristics of chronic and acute trials examining the effect of cocoa flavanols in patients with type 2 diabetes.

**References**	**Population (sex intervention group)/(sex control group)**	**Medication**	**Intervention**		**Control**		**Frequency and duration**	**Vascular assessment**
			**Form**	**Flavanol** ** content/d**	**Form**	**Flavanol content/d**		
**Chronic trials**								
Ayoobi et al. (40)	T2DM (14F + 7M)/ (13F + 10M)	Oral anti-DM drugs only, no information on anti-HT drugs	30 g 84% dark chocolate	No information	No intervention	No intervention	1x/d, 8 w	SBP↓, DBP ↓, NO =, angiotensin II =
Balzer et al. (41)	T2DM (13F + 8M)/ (16F + 4M)	Insulin allowed, anti-HT drugs allowed	Cocoa powder + 250 mL water	3 × 321 mg FL, 3 × 57.8 mg EC	Cocoa powder + 250 mL water	3 × 25 mg FL, 3 × 4.5 mg EC, matched for theobromine and caffeine	3x/d, 30 d	FMD ↓, MAP =, HR =
Curtis et al. (42)	T2DM, postmenopausal (47F)/(46F)	Insulin allowed, anti-HT drugs allowed	2 × 13.5 g flavonoid enriched chocolate	850 mg flavan-3-ols, 90 mg EC	2 × 13.5 g placebo chocolate	Matched for macronutrient content	2x/d (lunch + evening), 52 w	SBP =, DBP =, MAP =, PP = (PP variability ↓), CCA-IMT =, PWV ↓, AI =, ACE =, NO =, ET-1 =
Dicks et al. (43)	T2DM + HT (10F + 7M)/(7F + 11M)	Oral anti-DM drugs only, anti-HT drugs allowed	5 × 0.5 g cocoa powder capsules	207.5 mg flavanols, 40.4 mg EC, 13.6 mg C	5 × 0.5 g pure microcrystalline cellulose	No flavanols	3 in morning, 2 in evening, 12 w	SBP =, DBP=
Mellor et al. (44)	T2DM (5F + 7M)/(cross-over)	Oral anti-DM drugs only, anti-HT drugs allowed	3 × 15 g high polyphenol chocolate, 85% cocoa solids	16.6 mg EC	3 × 15 g low polyphenol chocolate, no non-fat cocoa solids	<2 mg EC, matched for macronutrient content	3x/d, 8 w	SBP =, DBP =
Rostami et al. (45)	T2DM + HT (20F + 12M)/(16F + 12M)	Oral anti-DM drugs only, anti-HT drugs allowed	25 g dark chocolate, 83% cocoa solids	450 mg flavonoids	White chocolate	No flavonoids	1x/d, 8 w	SBP ↓, DBP ↓
**Acute trials**								
Balzer et al. (41)	T2DM (2F + 8M)/(cross-over)	Insulin allowed, anti-HT drugs allowed	Cocoa powder + 250 mL water	*High*: 963 mg FL, 203 mg EC, 50.8 mg C *Medium*: 371 mg FL, 78.9 mg EC, 19.7 mg C	Cocoa powder + 250 mL water	75 mg FL, 16.8 mg EC, 4.2 mg C, matched for theobromine and caffeine	1x/d, 1, 2, 3, 4, 6 h post-intake	FMD ↓
Basu et al. (46)	T2DM + obese, (14F + 4M)/(cross-over)	Oral anti-DM drugs only, anti-HT drugs allowed	Cocoa powder + warm water, intake with a high-fat breakfast	480 mg FL, 40 mg EC, 18 mg C	Flavanol-free placebo powder + warm water, intake with a high-fat breakfast	<0.1 mg FL, not matched for theobromine or caffeine	1x/d, 30 min, 1, 2, 4, 6 h post-intake	SBP =, DBP =, large artery elasticity ↓, small artery elasticity =
Mellor et al. (47)	T2DM (1 postmenopausal F + 9M)/(cross-over)	Oral anti-DM drugs only, no information on anti-HT drugs	13.5 g high polyphenol chocolate + 200 mL water	3.5% polyphenols	13.5 g low polyphenol chocolate	0.9% polyphenols, identical formulation as intervention chocolate	1x/d, 3 h post-intake	Reactive hyperemia peripheral arterial tonometry ↓, endothelial serum adhesion molecules =

*The exact quantity of flavanols and/or epicatechin of the supplement is indicated only when this information is available in the paper; Effects in intervention group are expressed through: ↑, significant increase; ↓, significant decrease; =, no change; ACE, angiotensin-converting enzyme; AI, augmentation index at 75 heart rate; BP, blood pressure (systolic SBP, diastolic DBP); C, catechins; CCA-IMT, intima-media thickness of the common carotid artery; d, day; EC, epicatechins; ET-1, endothelin-1; F, female; FL, flavanols; FMD, flow-mediated dilation; h, hours; HR, heart rate; HT, hypertension; M, male; MAP, mean arterial pressure; min, minutes; NO, nitric oxide; PP, pulse pressure; PWV, pulse wave velocity; T2DM, Type 2 Diabetes Mellitus; w, weeks; y, year*.

The first step for profoundly testing these possible confounding factors would lie in the design of acute protocols applied to several well-characterized (particularly hypertension and its medications) groups of patients with T2DM. Up to now, three studies evaluated the vascular effects of acute CF supplementation in patients with T2DM (41, 46, 47). Two detected a significant improvement of endothelial function (41, 47) and one indicated a decrease in large artery elasticity (46) (Table 1). However, the same way as in the chronic studies, the three acute studies either gave no information about antihypertensive drugs or allowed the use of antihypertensive treatment (different types are reported) without considering this treatment as a possible confounding factor in the analyses (Table 1). To our knowledge, only four studies investigated the effect of CF when combined with antihypertensive drugs (different types are reported) in non-diabetic hypertensive adults (50, 51), in non-diabetic heart transplant recipients (52), and in non-diabetic adults with congestive heart failure (53). These showed a supplementary effect of cocoa intake on BP (50, 51) and/or endothelial function (50, 52, 53).

In addition, together with the heart, the micro- and macrocirculation determine the hemodynamics of the circulating system (54). It is important to study both systems simultaneously, but apart from one report (46) none of these studies in patients with T2DM investigated the effect of CF simultaneously in both of these vascular beds (i.e., micro and macrovascular beds) (Table 1).

In this study, we will take into account these results, assumptions, and points of heterogeneity as will be explained in this paper. In addition, this study will investigate the impact of CF on both micro- and macrovascular functions. As CF would increase nitric oxide, we hypothesize more effect on macrovessels as microvessels are also dependent on other vasodilators like prostaglandins. However, as little is known so far, our research will provide novel insights on this matter. Moreover, acute protocols will help to be sure of the efficacy of the dose chosen, to know which of the vascular (either micro or macrovascular) beds would be more impacted, and to identify whether some patients would be non-responders because of their medications. Hence, since our study has a robust methodology, it may act as a sort of “pilot” for the setup of long-term trials.

This acute, randomized, double-blinded, placebo-controlled cross-over study aims to investigate whether a single intake of a high dose of CF (790 mg flavanols) induces an improvement in endothelial function (primary outcome), a reduction in BP, and an enhancement in muscle vasoreactivity and oxygenation in patients with T2DM compared to healthy controls.

Because hypertension is a common comorbidity in patients with T2DM and as little is known so far concerning possible interferences between antihypertensive drugs and CF actions especially in patients with diabetes mellitus, this study will additionally investigate possible influence of beta-blockers (BB), angiotensin converting enzyme inhibitor (ACEi), or angiotensin receptor blocker (ARB) in diabetic persons with arterial hypertension and non-diabetic persons with essential hypertension treated with these drugs.

## Methods and Analysis

### Study Setting and Organization

The trial is a single center study and will be executed at the Ghent University Hospital (Belgium). All participants will complete the same protocol on both test days, however consuming a different type of capsules in a randomized order: high-flavanol cocoa extract or placebo. The flavanols used in this trial were extracted from the cocoa bean. A wash-out period of minimal 3 days and maximal 2 weeks is provided. The first measurement was conducted on October 5th, 2018 and since the COVID-19 pandemic forced to cancel all measurements for about 6 months, the end date of the study was postponed and is estimated in June 2021.

### Participants

#### Eligibility Criteria

All subjects are divided in four groups: (i) patients with T2DM with arterial hypertension (with BB, *n* = 5/ACEi, *n* = 5/ARB, *n* = 5) (*n* = 15), (ii) patients with T2DM without arterial hypertension (*n* = 10), (iii) non-diabetic patients with essential arterial hypertension (BB, *n* = 10/ACEi, *n* = 10/ARB, *n* = 10) (*n* = 30), and (iv) healthy participants (control group; *n* = 20).

Inclusion and exclusion criteria are summarized in Table 2.

**Table 2:** In- and exclusion criteria for participants in this study.

**Inclusion criteria**
Male and female; age: 18–85 years; BMI: 20–40 kg/m^2^
(i) Patients with T2DM without arterial hypertension: at least 5 years T2DM [HbA1c ≥6.5%, glucose (fasting) ≥126 mg/dl, glucose (not fasting): ≥200 mg/dl, defined by the American Diabetes Association (ADA) (55)]
(ii) Patients with T2DM with arterial hypertension: at least 5 years T2DM [HbA1c ≥6.5%, glucose (fasting) ≥126 mg/dl, glucose (not fasting): ≥200 mg/dl, defined by the American Diabetes Association (ADA) (55)] AND at least 1 year arterial hypertension taking BB, ACEi or ARB (optionally combined with diuretics)
(iii) Patients with essential arterial hypertension: at least 1 year arterial hypertension taking BB, ACEi or ARB (optionally combined with diuretics), matched by age, sex, and BMI with subjects with T2DM
(iv) Healthy controls: taking no medication except for contraceptive drugs, matched by age, sex, and BMI with subjects with T2DM
**Exclusion criteria**
Smoking habits: current smoking; smoking history of more than 30 years or pack years are higher than years of smoking cessation
Alcohol consumption: more than 10 units per week
Additional systemic disorders: chronic inflammatory disease, active cancer
Microvascular complications: retinopathy, nephropathy, peripheral sensory(motor), or autonomic neuropathy
Macrovascular complications: cardiovascular and respiratory diseases: heart failure NYHA class 3 and 4, uncontrolled arrhythmias or angora, documented peripheral arterial disease or experienced heart attack, active or chronic recurrent vasculitis, severe to very severe chronic pulmonary diseases (GOLD stage III and IV)
Neurological diseases: cerebrovascular accident, transient ischemic attack, reversible ischemic neurological deficit or stenosis >50% by doppler
Other: important (and relevant) musculoskeletal disorders; factors that impede the execution of the dynamic handgrip exercise test; pregnancy; known cognitive impairment (such as dementia, intellectual disability), language barriers
Medication: medication directly influencing endothelial function except for insulin or antihypertensive drugs; nitric oxide-containing medication; phosphodiesterase type 5-inhibitors

*T2DM, type 2 diabetes mellitus; BMI, body mass index; BB, beta blocker; ACEi, angiotensin converting enzyme inhibitor; ARB, angiotensin receptor blocker*.

#### Recruitment and Screening

Subjects are recruited by endocrinologists and other medical specialists, general practitioners, dieticians and investigator's acquaintances. Flyers are distributed in the Ghent University Hospital and by pharmacists and physiotherapists. In addition, the essential information for possible participants of our study is disseminated on media and social media.

#### Patient and Public Involvement

No patient or patient advisor was/is involved with study design, recruitment or conduct.

### Intervention

#### Study Protocol

**Figure 1:**
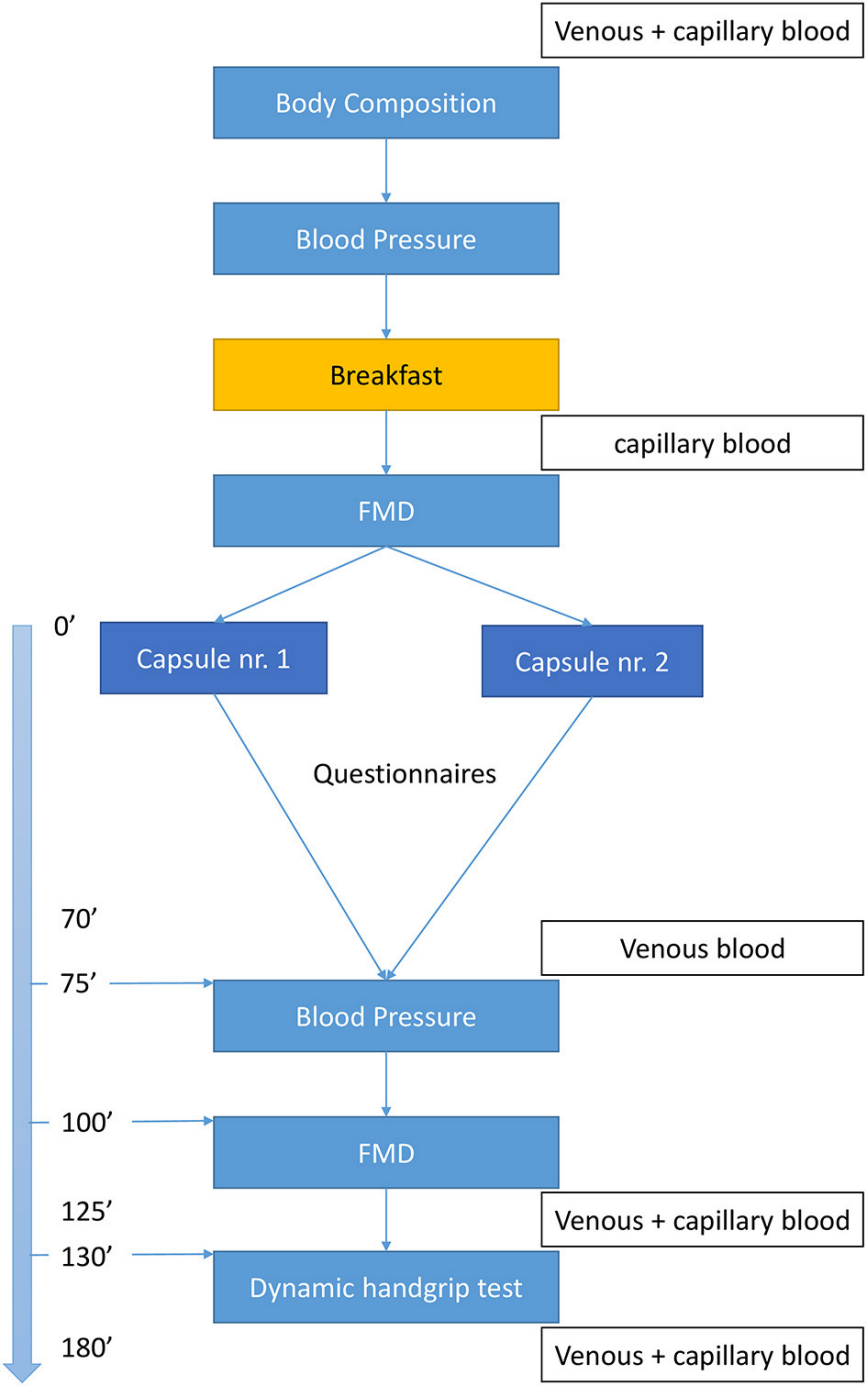
Flowchart. FMD, flow-mediated dilation test; capillary blood, finger prick to measure capillary glycaemia (only patients with T2DM).

As depicted in Figure 1, patients will arrive at the lab (Ghent University Hospital) at 8 o'clock after an overnight fast of minimal 8 h. Blood samples will be collected and participants will be evaluated for body composition [weight, height, skinfolds, and fat% (bio-electrical impedance analyzer)] and BP, followed by a standardized breakfast with negligible flavonoid and nitrate/nitrite amounts, in accordance to guidelines of a dietician (Table 3), and consumed within 15 min. Participants have to choose 1 formula of breakfast which will be consumed at both visits. After 15 min rest period, a baseline Flow-Mediated Dilation (FMD) measurement will be performed, followed by the ingestion of CF-enriched capsules (2.5 g of cocoa extract which contains 790 mg flavanols of which 150 mg epicatechin, *Naturex*^©^, *France*) or placebo (maltodextrin and an equivalent dose of theobromine and caffeine compared to the CF-enriched capsules) (Table 4). The dose of CF given in this study is based on the research of Balzer et al. (41) who investigated the effect of different doses of CF (high dose = 963 mg CF of which 203 mg epicatechin, medium dose = 371 mg CF of which 78.9 mg epicatechin, and low dose (control) = 75 mg CF of which 16.8 mg epicatechin), closely matched for theobromine and caffeine, on FMD in 10 patients with T2DM. The equilibration of theobromine and caffeine, both vasoactive compounds of cocoa (38, 56, 57), in the different interventions is a very important quality of this study and will ensure to test isolated vascular effects of CF.

**Table 3:** Standardized breakfast formulas.

**Breakfast formulas**	**Composition**	**Ingredients**
Formula 1	Energy: 418 Kcal Proteins: 21.7 g (21%) Carbohydrates: 61.4 g (59%) Fat: 8.8 g (20%)	60 g cereals (Special K, Kellogg's) 200 g semi-skimmed milk 125 g semi-skimmed cottage cheese
Formula 2	Energy: 386 Kcal Proteins: 19.3 g (21%) Carbohydrates: 49.5 g (53%) Fat: 10.8 g (26%)	90 g light brown bread 15 g butter (Halvarine, Blue Band) 17 g cream cheese (La vache qui rit) 15 g jam (reduced sugars) 125 g low-fat yogurt
Formula 3	Energy: 499 Kcal Proteins: 23.3 g (19%) Carbohydrates: 53.9 g (44%) Fat: 20.0 g (37%)	90 g light brown bread 200 g semi-skimmed milk 15 g butter (Halvarine, Blue Band) 15 g jam (reduced sugars) 30 g cheese (Gouda, Hollandic)
Formula 4	Energy: 501 Kcal Proteins: 22.4 g (18%) Carbohydrates: 58.6 g (48%) Fat: 17.6 g (33%)	90 g light brown bread 15 g butter (Halvarine, Blue Band) 25 g gingerbread (reduced sugars) 30 g cheese (Gouda, Hollandic) 125 g low-fat yogurt

**Table 4:** Nutrient content of the capsules.

**Nutrient content**	**8 CF-enriched capsules**	**6 capsules with placebo**
Total cocoa extract (g)	2.5	0
Total flavanols (mg)	794	0
Epicatechin (mg)	149	0
Catechin (mg)	30	0
Caffeine (mg)	23	24
Theobromine (mg)	179	180
Maltodextrin (mg)	928	1.956

*Amount is presented for total amount taken and not for each capsule. CF, Cocoa flavanols*.

The investigator will ensure all capsules are taken in properly. After ingestion, the participants will remain seated for 70 min. Thereafter, blood samples will be collected and a second BP and FMD assessment followed by a dynamic handgrip exercise (dHGE) test with simultaneous near-infrared-spectroscopy (NIRS) monitoring and blood flow measurements will be executed. Venous blood samples will be drawn before and after the dHGE test.

All measurements post intake will be performed within 2 h as highest circulating concentrations of flavanols are found between 90 and 120 min after consumption (58, 59).

Participants will fill in questionnaires (once) to evaluate factors which may impact vascular reactivity [i.e., daily physical activity [International Physical Activity Questionnaire—long version (60)], flavanols-intake [a self-designed questionnaire about the frequency of flavanol-enriched food intake (16, 17, 61)], sleep behavior (Epworth Sleepiness Scale, STOP-BANG), and autonomic function (Autonomic Symptom Profile-COMPASS). We will also assess quality of life and general health status (36-Item Short Form Health Survey, World Health Organization Quality of Life questionnaire-BREF, and only for patients with T2DM Diabetes Quality Of Life questionnaire). In addition to the questionnaires, each participant's physical activity levels (62) and glycemic excursions and variability (63) will be objectively measured during 1 usual week using accelerometry and a Continuous Glucose Monitoring System, respectively.

#### Guidelines for Participants

Subjects will be asked not to participate in other trials from 3 weeks before start of this study to 1 week after the second study day. Supplement or vitamin consumption that could interfere with the mechanisms of action of flavanols have to be suspended for at least 4 weeks prior start of the study. In addition, to minimize flavanol intake before each study day, participants will be asked to follow guidelines (16, 17, 61):

➣ 3 days before each study day, participants will be asked to drink maximal two cups of tea per day, to avoid red wine or cider, to refrain from eating chocolate (in any form), beans, and rhubarb and to consume maximal two small portions of fruits (piece, juice, or jam) or one portion in combination with 10 g nuts.
➣ Twenty-four hours before each study day, participants will be asked to refrain from vigorous physical activity (apart from daily movements as climbing stairs, biking to the train station, walking to the car etc.), alcohol or caffeine containing drinks (e.g., coffee, cola, and tea). They have to consume the same meal the evening before both study days.
➣ Eight hours before the actual start of the study, participants will be asked to fasten (no food or drink intake, apart a small amount of water) and, importantly, to take in their medication as usual (identical dose as prescribed by their physician). Antihypertensive medication need to be taken in exactly 2 h before actual start of the study. Antidiabetics must be taken in at home or at the lab during breakfast depending on type of the drug. Metformin, SGLT-2 inhibitors, and GLP-1 analoga may be taken in at home or at the lab and all sulfonylurea drugs must be taken in at the lab during breakfast. For insulin, the long-acting insulin must be taken in at home and the short-acting insulin must be taken in at the lab during breakfast.

#### Blinding and Randomization

In this cross-over study, every participant will receive CF-enriched and placebo capsules. Randomization of capsules will be done by sealed envelopes and type of capsules will be indicated by number 1 or number 2. Each participant may choose 1 envelope at the first study day. Both types of capsules have an identical look and taste. Hence, participants will be blinded to their group allocation. Furthermore, since type of capsules will be identified by numbers, outcome assessors and personnel involved in data collection and data analysis will be blinded to participants' group allocation throughout the entire trial.

#### Data Management

All researchers, outcome assessors, data collector, data manager, data entry personnel, and statistician will receive special training regarding the standard procedure and data management. During the recruitment period, our data collector will record the baseline characteristics of participants in case report forms and all data will be checked by the data manager.

Study data will be pseudonymized, collected, and managed using REDCap, a secure, GDPR-proofed, web-based software platform designed to support data capture for research studies, providing (1) an intuitive interface for validated data capture, (2) audit trails for tracking data manipulation and export procedures, (3) automated export procedures for seamless data downloads to common statistical packages, and (4) procedures for data integration and interoperability with external sources (64, 65).

Solely two main investigators (endocrinologist and study responsible) will have the authority to decode the pseudonymized data. Concerning obtained data of FMD measurements, all clips will be saved in dicom-format and stored at a central server (University Ghent).

#### Adverse Events and Safety Monitoring

Adverse events after intake will be described as it is monitored by oral interrogation during the study day and by e-mail if participants experience some side-effects. So far, no adverse effects were defined in literature. However, in the article of Monagas et al. (66) 1 out of 42 included subjects reported constipation during the intake of cocoa concomitant with 250 mL skim milk for 4 weeks, which was solved by increasing fiber intake.

### Outcome Measures

#### Primary Outcome Measure

##### Flow-Mediated Dilation

FMD is a non-invasive technique and the gold standard to measure endothelial function. The FMD measurement will be performed at the brachial artery, at the non-dominant side, following a standardized protocol described by Thijssen et al. (67). An ultrasound system (GE, Vivid 7) with a linear probe (12L, 7–10 MHz) will be used to assess the brachial artery diameter based upon longitudinal images with the lumen–intima interface visualized on both (anterior and posterior) walls. The arterial occlusion will be performed *via* a sphygmomanometer cuff (Hokanson, SC5™, 6 × 83 cm), placed 5–10 cm below the elbow, inflated and held between 220 and 240 mmHg (68, 69) for 5 min. For stability, the probe will be hold tight by a stand-off probe support. To assess the effect of the reactive hyperemia, the brachial artery will be visualized pre-occlusion for 90 s (mean of three individual clips comprising each 30 s) and post-occlusion for 4 min with additional 15 s before cuff release. Although it is recommended to perform an FMD measurement in fasting state (67), this is not feasible since the entire protocol lasts around 5 h. However, the measurements will be done 15 and 150 min after food intake (standardized breakfast at each visit). Boundaries for diameter measurement will be identified automatically by means of a boundary tracking software (Quipu, diastolic phase) and optically controlled by a single, independent and blinded investigator. When tracking is impaired, the investigator will restore this tracking with maintenance of region of interest. Data obtained from false tracking or altered region of interest will be removed from analysis. FMD measurement is a challenging technique and has a significant learning curve. At least 100 scans supervised by a specialist were done prior to the start of this study.

#### Secondary Outcome Measure

##### Blood Pressure

BP measurements will be carried out at the dominant side, in sitting position, after 3 min of rest by an automatic device (Tango+, SunTech Medical). Heart rate and systolic and diastolic BP will be assessed for 21 min with an interval of 3 min, pre- and post-capsules intake. During the recording, participants will be asked to remain calm and silent and refrain from drinking. Systolic and diastolic BP, as well as mean arterial pressure, pulse pressure, and heart rate will be used in analysis.

##### Dynamic Handgrip Exercise Test With Near-Infrared-Spectroscopy

A maximal dHGE will be performed with simultaneous NIRS (OxiplexTS, ISS, Champaign, IL, USA) monitoring to assess the oxygenation and vasoreactivity at the level of arterioles and capillaries of Musculus flexor digitorum superficialis, Musculus flexor carpi ulnaris, and Musculus flexor carpi radialis. A reliable handgrip exercise protocol, specifically designed for patient populations using NIRS measurements, will be carried out according to Celie et al. (70). In order to minimize bias and to work with relative values, an arterial occlusion of the ipsilateral upper arm will be performed by a pneumatic cuff (Hokanson, SC5™, 6 × 83 cm) inflated and held between 220 and 240 mmHg for 5 to maximal 7 min, until maximal deoxygenation (hemoglobin and myoglobin) values are reached (71). In this study, the maximal voluntary contraction will be calculated by means of two attempts with 10 s in between. The exercise test, consisting of 2-min periods of an incremental cyclic contractions protocol (1 s contraction, 1 s relaxation) followed by 1-min rest periods, will start at 20% of the maximal voluntary contraction and increase by 10% of the maximal voluntary contraction each step until exhaustion. Total hemoglobin [sum of deoxy- and oxyhemogblin, which reflects the change in regional blood volume (72)], deoxyhemoglobin and –myoglobin, and saturation will be assessed according to Celie et al. (70).

Blood flow through the brachial artery *via* echo-doppler will be measured according to Celie et al. (71) (same transducer and linear probe as used for the FMD measurement) at baseline (before cuff occlusion), immediately after cuff occlusion, and in the periods of rest after each increasing step.

##### Blood Analyses

Levels of uric acid (photometry, Architect c16000, Abbott), vitamin C (colorimetric method, R-biopharm/Roche reagent, Indiko Plus, Thermo Fisher), and glucose (photometry, Architect c16000, Abbott) will be measured at each study day in whole blood (anticoagulant-free tubes). Plasma and serum derived from EDTA and anticoagulant-free tubes will be centrifuged (3,500 g for 10 min at 10°C) and thereafter stored at −80°C together with a cryovial whole blood in the medical biobank of the Ghent University Hospital (Bioresource center Ghent, Gent, Belgium, ID: BE 71067049) (73). At the end of the study, cholesterol (photometry, Architect c16000, Abbott), high density lipoproteins (photometry, Architect c1600, Abbott), triglycerides (photometry, Architect c16000, Abbott), high sensitive C-reactive protein (particle-enhanced immunonephelometry, BN II, Siemens), vitamin E (liquid/liquid extraction followed by detection *via* UPLC-DAD), free fatty acids (enzymatic colorimetry, Cobas 8000 e801, Roche Diagnostics), and free insulin (Cobas e801 Roche, ECLIA) will be determined in serum, while hemoglobin A1c (exchange chromatography—Tosoh HLV-723 G8) and haptoglobin (Behring Nephelometer analyzer II), a marker of iron metabolism (74), will be determined in whole blood. All samples will be analyzed at the lab of clinical biology of Ghent University Hospital.

#### Additional Outcome Measures

In each participant, physical activity [steps per day and moderate-to-vigorous physical activity (MVPA)] will be evaluated with an accelerometer [Actigraph wGT3X-BT, dominant side, hip (75)] and glycemic excursions (percentages of time in hyper- or hypoglycemic ranges and time in range), risk for hypo- and hyperglycemia (Low and High Blood Glucose Indexes, LBGI and HBGI), and glycemic variability [standard deviation, coefficient of variation, and mean amplitude of glucose excursion (MAGE)] (76) by continuous interstitial glucose measurement (blind mode, IPro2, Medtronic, abdomen) for 1 week, immediately after the second study day. During this week, the above-mentioned restrictions concerning food intake and physical activity are not applicable and participants are encouraged to maintain their normal daily habits. Additionally, participants are asked to fill in a diary concerning their food intake and their physical activities for each day.

### Statistical Analyses

#### Power and Sample Size Calculation

Sample size calculations are based on previous research focusing on interventional studies with FMD measurements (77, 78) who established that in cross-over trials at least 20–30 participants are required for a minimal detectable change of 1.5–2% in FMD (80% power, alpha-level = 0.05). Sample size calculation was done by SAS power and sample size, indicating a minimum of nine participants in each group (41). Data will be analyzed taking into account volunteer- and methodology-related factors (79).

#### Data-Analysis Plan

Effects of CF ingestion on primary and secondary outcome measures will be evaluated within and between groups. Data will be analyzed using a random intercept model in mixed models. Fixed effects will be the group, the supplementation (cocoa flavanols vs. placebo), the time (pre- vs. post-intake of capsules) during each visit for repeated measurements, as well as group × supplementation interaction and time × supplementation interactions. We will also consider the order of both visits in a first intent and this factor will be kept in analyses only if significant. As all groups are matched by age, sex, and BMI, only medication intake, level of physical activity, glycemic excursions, risk for hypo- or hyperglycemia, and glycemic variability will be considered as covariates when analyzing data. In case of drop-outs or missing data, the participant is still included in data analysis providing one out of two visits is completed.

Results will be presented as mean ± standard deviation with its corresponding 95% confidence interval. Level of significance will be set at *p* < 0.05. Data analysis will be conducted using IBM SPSS statistics version 26.

## Discussion

In recent years, there has been an increased attention to polyphenols and their beneficial effects on vascular health. Several studies have been carried out in healthy participants to assess this. However, the studies, acute as well as chronic, on patients with T2DM with a high risk for vascular complications are scarce and show inconsistent results. Possible reasons for this inconsistency might be heterogeneity in the given intervention (dose, duration, source), the studied population (duration of diabetes, severity of comorbidities, BP at baseline, sex), and possibly used medication (vasoactive antihypertensive and antidiabetic drugs). Hence, there is a high need for more acute and chronic research concerning this topic in this population.

This work will provide novel data helpful for the development of strategies in the nutritional education of particularly vulnerable populations, given their high risk for developing cardiovascular disease, including non-pharmacological therapies and strategies that employ lifestyle modification. This intervention might also have implications for the preparation of recommendations in clinical practice guidelines and quality improvement programs aimed at the care of patients with T2DM.

## Ethics Statement

Study procedures were approved by the ethical committee of Ghent University Hospital on April 5th, 2018 (STUDY B670201835660). To enquire all participants and to ensure making an informed decision to participate, each subject will receive an informed consent that first is explained orally by an investigator and thereafter sent by e-mail providing the opportunity to reread this form and to ask questions afterwards. The informed consent contains all details of the research (background, aims, and possible risks or advantageous) and is written in Dutch for good understanding. In case of important protocol modifications, all participants will be informed by e-mail. The results will be submitted to an international peer-reviewed journal and presented at scientific conferences. They will be disseminated through digital science communication platforms, including academic social media, to extend its outreach and usefulness.

## Author Contributions

AT, BC, SS, ER, JO, KR, EH, and PC were involved in the methodological design and drafting of the trial protocol. AT, SS, ER, PC, and EH have overall responsibility for the design, conduct, and decision to submit for publication. BC, JO, and KR are co-researchers. BC designed the dynamic handgrip exercise test and set up the plan for analysis. JO and KR designed the flow-mediated dilation measurement and set up the plan for analysis. AT, ER, SS, PC, and EH recruit participants into the study. AT, PC, and EH drafted the manuscript. All authors will contribute to data interpretation, conclusions, and dissemination and read and approved the final manuscript.

## Conflict of Interest

The authors declare that the research was conducted in the absence of any commercial or financial relationships that could be construed as a potential conflict of interest.

## Abbreviations

ACEi: angiotensin converting enzyme inhibitor
ARB: angiotensin receptor blocker
BB: beta-blockers
BP: blood pressure (SBP, systolic blood pressure; DBP, diastolic blood pressure)
CF: cocoa-derived flavanols
dHGE: dynamic handgrip exercise
FMD: flow-mediated dilation
NIRS: Near-infrared spectroscopy
T2DM: type 2 diabetes mellitus.
