# Supplementary material for: Acute Effects of Cocoa Flavanols on Blood Pressure and Peripheral Vascular Reactivity in Type 2 Diabetes Mellitus and Essential Hypertension: A Protocol for an Acute, Randomized, Double-Blinded, Placebo-Controlled Cross-Over Trial
Source: Front Cardiovasc Med. 2021 Mar 15;8:602086. doi: 10.3389/fcvm.2021.602086 (PMC8005536; doi:10.3389/fcvm.2021.602086)
Supplement: Supplementary file 1 [file Table_1.DOCX]

Supplementary Material

Table S1: Participant timeline

|  |  | Study day x* | | | Study day y* | | |  |
| --- | --- | --- | --- | --- | --- | --- | --- | --- |
|  | Enrollment | Pre-allocation | Allocation | Post-allocation | Pre-allocation | Allocation | Post-allocation |  |
| Time point | Before first study day | T _-1_ | T _0_ | T _1_ | T _-1_ | T _0_ | T _1_ | After second study day |
| Enrollment | | | | | |  |  |  |
| Eligibility screen | x |  |  |  |  |  |  |  |
| Informed consent | x |  |  |  |  |  |  |  |
| Interventions (depending on number in sealed envelope) | | | | | |  |  |  |
| Capsules with cocoa flavanols |  |  | x |  |  |  |  |  |
| Capsules with placebo |  |  |  |  |  | x |  |  |
| Assessments | | | | | |  |  |  |
| Baseline variables: Body mass index, venous blood sample |  | x |  |  | x |  |  |  |
| Flow-mediated dilation measurement |  | x |  | x | x |  | x |  |
| Blood pressure measurement |  | x |  | x | x |  | x |  |
| Dynamic handgrip exercise test with Near-infrared spectroscopy |  |  |  | x |  |  | x |  |
| Additional measurements: Accelerometer and continuous glucose monitoring system |  |  |  |  |  |  |  | x |

*: the intake of the capsules is randomized so study day x or study day y can be the first or the second study day.
